# Examining the synergistic effects of a cognitive control video game and a home-based, self-administered non-invasive brain stimulation on alleviating depression: the DiSCoVeR trial protocol

**DOI:** 10.1007/s00406-022-01464-y

**Published:** 2022-10-22

**Authors:** Esther Dechantsreiter, Frank Padberg, Alon Morash, Ulrike Kumpf, Arthur Nguyen, Zeno Menestrina, Fabienne Windel, Gerrit Burkhardt, Stephan Goerigk, Takuya Morishita, Aldo Soldini, Shira Ahissar, Tamar Cohen, Angela Pasqualotto, Linda Rubene, Liene Konosonoka, Daniel Keeser, Peter Zill, Razan Assi, Rémy Gardier, Roser Viñals, Jean-Philippe Thiran, Ronen Segman, Yuval Benjamini, Omer Bonne, Friedhelm Christoph Hummel, Daphne Bavelier, Elmars Rancans, Mor Nahum

**Affiliations:** 1 Department of Psychiatry and Psychotherapy, Ludwig-Maximilians-University Munich, Nussbaumstr. 7, 80336 Munich, Germany; 2 NeuroImaging Core Unit Munich (NICUM), University Hospital LMU, Munich, Germany; 3 Hadassah Medical Center, Jerusalem, Israel; 4 Faculty of Psychology and Educational Sciences, University of Geneva, and Campus Biotech, Geneva, Switzerland; 5 Defitech Chair of Clinical Neuroengineering, Neuro-X Institute (INX) and Brain Mind Institute (BMI), Ecole Polytechnique Fédérale de Lausanne (EPFL), Geneva, Switzerland; 6 Defitech Chair for Clinical Neuroengineering, INX and BMI, EPFL Valais, Clinique Romande de Réadaptation, Sion, Switzerland; 7 Department of Psychological Methodology and Assessment, Ludwig-Maximilians-University, Leopoldstraße 13, 80802 Munich, Germany; 8 Charlotte Fresenius Hochschule, Infanteriestraße 11A, 80797 Munich, Germany; 9 School of Occupational Therapy, Faculty of Medicine, The Hebrew University, Jerusalem, Israel; 10 Department of Psychiatry and Narcology, Riga Stradins University, Riga, Latvia; 11 Riga Centre of Psychiatry and Addiction Disorders, Riga, Latvia; 12 Signal Processing Laboratory 5 (LTS5), Ecole Polytechnique Fédérale de Lausanne (EPFL), Lausanne, Switzerland; 13 CIBM Center for Biomedical Imaging, Lausanne, Switzerland; 14 Radiology Department, Centre Hospitalier Universitaire Vaudois and University of Lausanne, Lausanne, Switzerland; 15 Department of Statistics and Data Science, Faculty of Social Sciences, The Hebrew University, Jerusalem, Israel; 16 Department of Clinical Neurosciences, Geneva University Hospital (HUG), Geneva, Switzerland

**Keywords:** Major depressive disorder, Cognitive control, Video game, Transcranial direct current stimulation (tDCS), Home-treatment, Feasibility

## Abstract

Enhanced behavioral interventions are gaining increasing interest as innovative treatment strategies for major depressive disorder (MDD). In this study protocol, we propose to examine the synergistic effects of a self-administered home-treatment, encompassing transcranial direct current stimulation (tDCS) along with a video game based training of attentional control. The study is designed as a two-arm, double-blind, randomized and placebo-controlled multi-center trial (ClinicalTrials.gov: NCT04953208). At three study sites (Israel, Latvia, and Germany), 114 patients with a primary diagnosis of MDD undergo 6 weeks of intervention (30 × 30 min sessions). Patients assigned to the intervention group receive active tDCS (anode F3 and cathode F4; 2 mA intensity) and an action-like video game, while those assigned to the control group receive sham tDCS along with a control video game. An electrode-positioning algorithm is used to standardize tDCS electrode positioning. Participants perform their designated treatment at the clinical center (sessions 1–5) and continue treatment at home under remote supervision (sessions 6–30). The endpoints are *feasibility* (primary) and *safety*, *treatment efficacy* (secondary, i.e., change of Montgomery-Åsberg Depression Rating Scale (MADRS) scores at week six from baseline, clinical response and remission, measures of social, occupational, and psychological functioning, quality of life, and cognitive control (tertiary). Demonstrating the feasibility, safety, and efficacy of this novel combined intervention could expand the range of available treatments for MDD to neuromodulation enhanced interventions providing cost-effective, easily accessible, and low-risk treatment options.

ClinicalTrials.gov: NCT04953208.

**Supplementary Information:**

The online version contains supplementary material available at 10.1007/s00406-022-01464-y.

## Background

Major depressive disorder (MDD) is a highly prevalent psychiatric disorder [1–3], afflicting millions of people worldwide [4]. MDD has been ranked as a leading contributor to the global burden of disease [4] and is associated with a high risk for suicidal ideation and behavior [5]. Despite advances in psychopharmacological and psychotherapeutic treatments for MDD, significant limitations remain. Caveats include potential unfavorable side effects of pharmacotherapy [6, 7] and limited accessibility of psychotherapeutic care. A relevant proportion of MDD patients does not adequately respond to standard therapies and experience a recurrent or chronic course of illness [8–12]. Furthermore, new treatment approaches, e.g., transcranial magnetic stimulation or esketamine are mainly delivered in specialized settings significantly reducing their accessibility. Therefore, further safe, and cost-effective treatment strategies are still needed, ideally expanded as clinically supervised treatments to the patient’s home environment.

Cognitive models of depression suggest that deficits in cognitive control (CC), i.e., the mechanisms underlying one’s goal-directed behavior while inhibiting task-irrelevant information, are key, and potentially causal factors in MDD [13–16]. In the past, CC deficits have been mainly considered as accompanying phenomena in MDD but have more recently been proposed as a psychological core factor for MDD vulnerability [14, 15, 17]. Indeed, CC dysfunction is frequently reported by patients suffering from MDD, associated with perseverative depressive rumination [18] and reflected by decreased activity in prefrontal brain areas [19, 20]. Similarly, overall MDD symptomatology has been associated with a hypoactivation of lateral prefrontal brain areas [21], rendering methods targeting prefrontal neural activity especially promising to alleviate MDD symptoms in general and cognitive control deficits in particular. Approaches hypothesized to alter prefrontal neuronal activity include prefrontal transcranial direct current stimulation (tDCS) as has previously been demonstrated in analogy to motor cortex studies [22–24], and various behavioral forms of CC training (CCT).

Several randomized controlled trials [25–27], as well as a recent meta-analysis [28], suggest that tDCS exerts clinically relevant antidepressant effects. Yet, negative results [29] emphasize the need for further confirmatory trials to establish its efficacy as a monotherapy. An alternative, or complementary, approach is that of CCT, designed to specifically enhance the plasticity-based mechanisms associated with CC function [30]. Similarly to tDCS, short-term CCT, involving either working memory (WM)-based or inhibitory control training paradigms, was found to improve CC function and alleviate depressive symptoms [31–33]. However, other studies reported only limited antidepressant efficacy of CCT [34–39]. A recent meta-analysis [40] found a small effect (Hedge’s g = 0.28) of CCT on depressive symptoms. Interestingly, studies that used emotional, rather than neutral CC-based training tasks, found more significant effects on mood and depression [41–44], potentially due to the activation of CC mechanisms through emotional stimuli.

These encouraging, yet somewhat mixed results call for innovative treatment strategies, potentially combining the effects of single interventions into multi-modal synergy-based or enhanced approaches (e.g., neuromodulation enhanced cognitive training or psychotherapy) to boost their antidepressant effects (see [45]). Within the scientific field of non-invasive brain stimulation (NIBS), synergy-based approaches have combined tDCS with antidepressant medication [26, 46], cognitive behavioral therapy (CBT) [47, 48] and with CCT [49–53]. Our approach in the current project is to combine tDCS with an action-like video game as a variant to CCT, exploiting the potential of action video games in enhancing cognition through attentional control training, which is proposed to target the fronto-parietal attention network [54, 55]. In combination, tDCS and gamified CCT might ideally engage prefrontal subregions and circuits to alleviate rumination and depressive symptoms [56]. Since both, tDCS and game play can be delivered in an easily accessible, cost-effective manner even as treatment at home, this intervention may be particularly promising for widely scalable therapeutic interventions in the field of depression.

The aim of this clinical randomized controlled trial (under the acronym DiSCoVeR: Depression Stimulation Cognitive Control Video game Remotely) is to evaluate the feasibility, safety, and efficacy of a novel treatment approach for MDD, synergistically combining tDCS along with an action-like video game, applied remotely from home. The custom-made video game, Legends of Hoa’manu (LoH), is designed to include both action video game mechanics, shown to enhance attentional control [57], as well as CCT exercises, with the goal of enhancing executive functions. By extending available interventions to the private home setting, the proposed therapeutic approach could help increase the accessibility of MDD interventions. We further examine mechanisms of change and endurance of effects following treatment completion. We hypothesize that the combined intervention will be feasible and safe. Furthermore, as a secondary endpoint, we expect a larger therapeutic effect on depressive symptoms for the combined active treatment compared to the control intervention.

## Trial design and methods

### Trial design

The DiSCoVeR clinical trial is a multi-center, two-arm, double-blind, randomized controlled trial (RCT), conducted at three clinical sites (Hadassah Medical Center, Israel; Riga Stradins University, Latvia; Ludwig-Maximilians-University, Germany). The goal of the trial is to examine the feasibility and safety, as well as, secondarily, the efficacy of a combined treatment approach (tDCS + LoH video game) with components that have been developed and integrated in the preceding phases of the DiSCoVeR project.

**Fig. 1:**
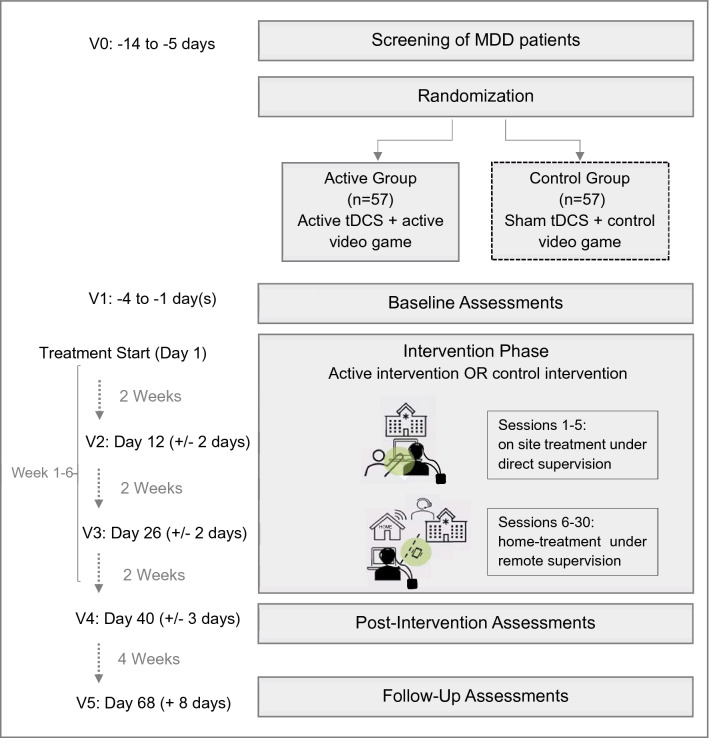
Study flow chart

*Study visits* are scheduled for screening (V0) and at baseline (V1), at two time points during the intervention (week 2, V2; week 4, V3), at week 6 upon completion of the intervention (V4), and at week 10 at follow-up (V5) (Fig. 1). All patients are required to provide written informed consent prior to participating in any study-related procedures. During the *screening phase,* patients are evaluated for study eligibility based on the inclusion and exclusion criteria described below. Eligible patients are randomly assigned to one of two study arms: (A) active condition (active tDCS + LoH game) or (B) control condition (sham tDCS + LoH_control game). In the *baseline phase*, data are obtained using clinical rating scales, self-rating questionnaires, and CC tasks.

During the *intervention phas*e, both treatment conditions are carried out for a duration of six weeks. In total, 30 treatment sessions are performed daily during weekdays for 30 min per day (15 h of intervention in total). This dose is similar or higher compared to doses used in previous antidepressant trials of tDCS [26, 27, 46] and of CCT [15, 31, 41]. In case of missed treatment sessions, up to four sessions may be compensated for on weekends and up to four sessions may be compensated for in week 7, following the post-intervention assessment after week 6.

The *treatment plan* consists of an on-site period at the clinical center (sessions 1–5; week 1) followed by a home-based intervention under remote supervision (sessions 6–30; weeks 2–6). During the first week, study operators train patients on how to use the combined treatment device. Patients gradually transition to self-manage their treatment based on the instructions and continue from home during the remainder of treatment. On an individual basis, on-site treatment can be extended or abbreviated, based on both the patient's and investigator’s confidence that the treatment can be self-administered at home.

For self-administered home-treatment, patients are supplied with an internet-enabled tablet including the pre-installed video game and tDCS software to support correct electrode positioning and an adequate application of tDCS. Patients are asked to maintain a constant training environment by adhering to the following conditions: adopting an upright sitting position on a chair, minimizing distracting factors such as phones or the presence of other people in the room, and a stable internet connection. Operators provide patients with remote support via telephone or video-call during the home-treatment phase on a per-need basis. In addition, patients are contacted at least once a week, to ensure that treatment is proceeding as planned, as well as to motivate patients for optimal compliance. In addition, study staff continuously monitor session parameters and session completion remotely via a dedicated clinician portal. For safety reasons, the activation of the treatment device by patients outside of the pre-scheduled time slots is disabled.

During the *follow-up phas*e, there are no restrictions in terms of antidepressant treatment strategies; yet any modifications in the treatment plan need to be documented.

The expected recruitment period amounts to approximately 20 months (recruitment rate of approx. 2–3 patients/month/study site).

### Study sites

The clinical trial is conducted at the following study sites: (1) Department of Psychiatry and Narcology, Riga Stradins University (RSU), Riga, Latvia (PI: E.R., MD), (2) Department of Psychiatry, Hadassah Medical School, Hebrew University, Jerusalem, Israel (PI: O.B., MD), and (3) Department of Psychiatry and Psychotherapy, University Hospital, LMU Munich, Germany (PI: F.P., MD). The trial is coordinated and monitored centrally by the coordinating study site (HUJI; PI: M.N.), supported by the co-coordinating study site (LMU; PI: F.P.).

The three clinical trial sites are in charge of patient recruitment, patient screening, data assessment, and implementation of the intervention. For this purpose, the sites are equipped with the treatment set-up and with comprehensive training materials, including manuals, instruction videos, and standardized operation procedures (SOPs). *Study operators* and *unblinded investigators* were trained for the use of the treatment device including its preparation, implementation, and dismantling. Their training included control of the integrated gaming and stimulation software including the correct use of the algorithm-controlled application for facilitated electrode positioning, on-site patient teaching of proper equipment handling, direct supervision of on-site treatment and remote supervision of home-treatment, as well as comprehensive and adaptively extended troubleshooting protocols. *Raters* (*blinded investigators*) underwent assessment training. Both unblinded investigators and raters were trained on how to use the electronic database.

Ongoing scientific and technological support during the trial is provided by the following project collaborators: Faculty of Psychology and Education Sciences, University of Geneva (UNIGE), Geneva, Switzerland (PI: D.B.) and Faculty of Medicine, Hebrew University of Jerusalem (HUJI), Jerusalem, Israel (PI: M.N.) for the use of the LoH and LoH_control video games; Ecole Polytechnique Fédérale de Lausanne (EPFL), Switzerland (PI: F.C.H.) for the use of the tDCS device and the algorithm-controlled electrode positioning software. Additional technical support for the use of the tDCS device is given by the support team of Neuroelectrics Inc..

### Participants

Men and women, 18–65 years of age, with a primary diagnosis of an unipolar major depressive episode (single or recurrent) according to the DSM-5 criteria are included in the study. The duration of the current depressive episode should range between four weeks and five years. Current depressive episodes are delineated from previous ones by a period of ≥ 2 months during which the patient did not fully meet the DSM-5 definition of MDD. Depression severity at study inclusion should be a total score of ≥ 13 on the Hamilton Depression Rating Scale (HDRS-17; [58]). Patients without antidepressant medication or patients on a stable (i.e., for at least 4 weeks) dose of pre-defined medications (see Online Resource 1 for details) as monotherapy or in combination may be included. Concomitant psychotherapy is allowed with its type and frequency documented throughout the study course. Only patients that are capable and willing to provide informed consent are enrolled in the study.

Exclusion criteria include the following: participation of site personnel and investigators, directly affiliated with this study, and their immediate families; addiction to gaming; individuals diagnosed with any relevant psychiatric axis-I and/or axis-II disorders other than MDD as assessed by the *Mini-International Neuropsychiatric Interview* (*M.I.N.I.*; [59];) patients diagnosed with a significant neurological disorder; history of electroconvulsive therapy (ECT) treatment in the current episode; history of tDCS, except for single tDCS sessions during experimental studies; use of any investigational drug within 6 weeks from baseline; suicidal risk (a score of 4–6 on item 10 of the *Montgomery and Åsberg Depression Rating Scale* (*MADRS*; [60]) or attempted suicide in the current episode; intracranial implant or any other metal object within or near the head (excluding the mouth) that cannot be safely removed; implanted neurostimulators; known or suspected pregnancy (according to pregnancy test); history of seizures; treatment with deep brain stimulation or vagus nerve stimulation and/or any other intracranial implants (clips, cochlear implants); any relevant unstable medical condition (e.g. acute, unstable cardiac disease).

### Interventions

**Fig. 2:**
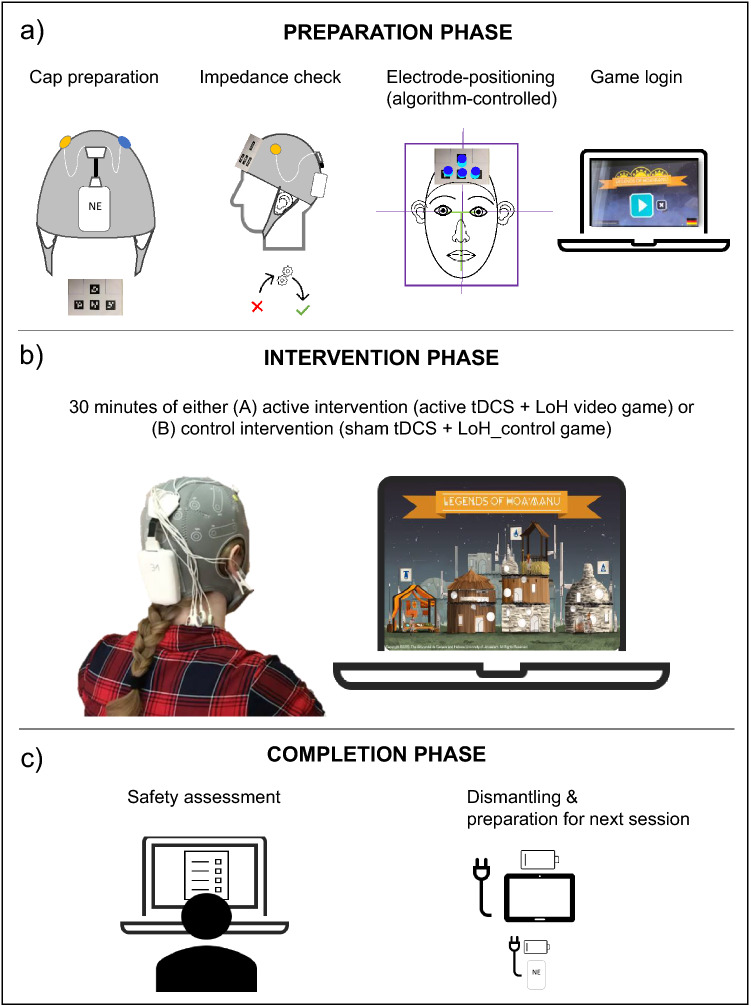
Treatment procedure. **a** Preparation: (from left to right): electrodes are inserted in saline-soaked sponges and are clipped into pre-defined holes (F3/F4) in the neoprene cap. The stimulator is attached to the back of the cap. The marker card displaying four reference points is attached to the front of the cap and will be used for the subsequent electrode positioning. After the cap is placed on the head, an ear clip is attached for grounding; electrode impedance is checked before session initiation; algorithm-controlled electrode positioning is applied for correct cap placement: patients place their heads within a reference frame visible on the tablet screen. A T-shape appears above key facial positions. Dots visible on the screen should be located above the reference points of the marker card. Real-time visual feedback is provided by the algorithm to indicate whether the cap is placed accurately or whether corrections are required. Game-login requires the patient’s study ID and a password. **b** Intervention: 30 min of active or control intervention. Session information is synchronized in real-time with the NE portal. Email notifications are provided to unblinded staff in case of stimulation events (e.g., start or end of session, session interruptions). **c** Completion: patients fill in a safety evaluation form. Unblinded staff are notified via email about the responses. Patients dismantle and clean the set-up and prepare it for the next session by recharging the stimulator and the tablet

Patients are randomized to receive either active tDCS + LoH video game or sham tDCS + LoH_control video game for 6 weeks. The treatment procedure is shown in Fig. 2.

### Transcranial direct current stimulation (tDCS)

The tDCS protocol follows similar protocols applied in previous clinical trials in depression [26, 46]. A treatment period of 6 weeks has been chosen based on findings suggesting that an extended treatment period may lead to a better response [27]. Stimulation is conducted using the tDCS 5G kit from Neuroelectrics (NE; Spain). A pre-installed user interface on a provided tablet (Microsoft Surface Go), the NE home-application, guides patients through the stimulation session and is synchronized with the video games. Stimulation scheduling and management is performed remotely by the unblinded investigators and study operators using a dedicated portal (NE portal; provided by Neuroelectrics) as has been suggested before [61].

Active tDCS starts with a fade-in phase (15 s), followed by a stimulation over 30 min with a stimulation intensity of 2 mA and a current density of 0.08 mA/cm^2^, and ends with a fade-out phase (30 s). Sham tDCS starts with a fade-in phase (15 s), followed by a 2 mA plateau (5 s), a fade-out phase (30 s), 28 min and 55 s of pause, and ends with a fade-in phase (30 s) and a fade-out phase (30 s). A bifrontal electrode montage with two 25cm^2^ round rubber electrodes inserted in sponges is used. The anode is attached over the left dorsolateral prefrontal cortex (DLPFC; center over F3 according to the international 10–20 EEG System). The cathode is placed over the right DLPFC (center over F4) using a neoprene electrode cap.

Ongoing work (Windel et al. in preparation) suggests that correct electrode placement can be ensured and monitored across treatment sessions using a novel electrode-positioning algorithm. The algorithm captures a set of markers placed on a neoprene cap holding the stimulation electrodes, as well as individual facial key points. During the first on-site treatment session, patient-specific cap positioning is performed by the trained investigators. For this purpose, the cap is placed on the patient’s head using the international 10–20 EEG system guidelines [62]. The patient’s individual facial key points are measured and saved with respect to the cap position as a reference. This reference is subsequently used during the verification process of the cap positioning in all following treatment sessions. The real-time visual feedback provided by the algorithm either indicates correct placement of the cap to the patients or instructs them to correct the placement toward the reference position.

### Experimental video game and control video game

During each stimulation session, patients engage in either an active or a control video game for the entire 30 min session. Both games are played on the supplied tablet (Microsoft Surface Go) with an external keyboard and are synchronized to the tDCS session via the home application. Games require a password-protected login, provided centrally by the study staff. Data from each game session are transferred to a secure server and are reviewed and monitored centrally by HUJI. During game play, patients are constantly updated by displaying information about their progression through various tasks of the game.

The experimental game (Legend of Hoa'manu; LoH) builds on a game architecture that combines action video game play with mini games built to train CC. It has been shown to successfully enhance attentional control in children [63]. Specifically, action video game play is designed to simultaneously load on pacing, divided attention, and focused attention, constantly challenging the flexible allocation of cognitive resources as task demands shift. Such game mechanics appear central to the cognitive enhancement noted after action video game play [57]. In addition, the LoH video game includes other game play activities that train specific CC abilities, including inhibitory control, working memory, task switching, planning, sustained attention, divided attention, and multitasking. For the purpose of the DiSCoVeR project, the existing game has been modified to include not just CC tasks but also tasks that involve emotional stimuli, as well as reward mechanics likely to favor positive mood, in an effort to yield a more effective outcome related to depression symptoms (see, e.g., [41–43, 64, 65]).

The control game (LoH_control) was created for the DiSCoVeR project to provide a gameplay experience similar to the LoH video game; this is critical for maintaining blinding of study patients for the specific intervention arm. The LoH_control﻿ game includes four mini-games; however, these contain neither action mechanics nor emotional or cognitive control challenges. Rather, these tasks employ slow paced activities such as puzzle-like activities or ones based on the slow and deliberate movement of focused attention or visual search with low target prevalence. Similar activities have been used as control training in studies with depressed or dysphoric patients [15, 31, 49, 66].

The first mini-game consists of a flying game which presents the same esthetics as its action-like counterpart in the experimental game (LoH). However, here the player only uses one cursor to move the bird (Raku), and there are no enemies to eliminate or dodge but only resources to collect, limiting the presence of any action mechanics. In a second mini-game, the players have to manage islands; interacting with the game environment, they can place plants, pet animals, and collect resources. A third mini-game consists of a visual search task requiring the player to find four targets hiding in a highly cluttered map. The fourth mini-game is based on the adaptive peripheral vision task (PVT) [15] in which the player needs to indicate the color or the symbol contained in a circular array of disks. This task requires peripheral vision skills to move one’s attentional focus, but not the eyes, one disk at a time in a clockwise direction in response to repeated presentation of auditory ‘go’ cues. Importantly, this task has been used before as a control training by several authors [31, 49, 66, 67].

Both the LoH and LoH_control video games place the patients in an adventure world with tasks and subtasks while patients discover *the Village*, a location aimed at maintaining the patients` motivation by allowing them to buy accessories, clothes, and other esthetical upgrades for their character. Within each game, patients progress through each task adaptively based on algorithms that individualize progression to enhance their sense of self-mastery as well as their willingness to persist.

### Assessments

The full list of study assessments and their timing of administration during the trial is presented in the *Time and Events Schedule* (Online Resource 2). Adjunctive assessments include assessments of salivary predictors of treatment response and epigenetic modification during treatment and an imaging paradigm (Online Resource 3).

### Screening assessments

At screening, study eligibility is determined based on the inclusion and exclusion criteria listed above. To determine whether DSM-5 criteria for MDD are met, study participants are interviewed using the *M.I.N.I.* [59]. Depression severity at the time of screening is obtained using the *HDRS-17* [58]. The *Gaming Disorder Test* (*GDT*; [68]) is used to rule out a pre-existing gaming addiction based on a self-report about gaming-related activities performed during the past year. Basic demographic information, the patients’ general medical and psychiatric history, their current and concomitant treatment, prior antidepressant treatment attempts, and their family history of psychiatric illness are assessed. Additionally, a physical and neurological examination is conducted by a medical doctor. Additional assessments include handedness assessment (*Edinburgh Handedness Inventory*; EHI; [69]) and smoking status (the self-report *Fagerström Test for Nicotine Dependence; FTND*; [70]).

### Study assessments

*Feasibility* is continuously assessed during the intervention phase. After each treatment session, it is recorded whether the session has been completed successfully. Reasons for interruptions, discontinuation, and potential restarts of the device are also carefully documented.

*Depression severity* as a study endpoint is measured using the *MADRS* [60]. From the baseline visit (V1) to the follow-up visit (V5), the clinician-based MADRS rating is complemented by self-assessment measures of depressive symptoms including the *Beck Depression Inventory* (*BDI-II*; [71]) and the *Patient Health Questionnaire*; (*PHQ-9* [72]*;*). We additionally assess anxiety using the *Generalized Anxiety Disorder 7-item* (*GAD-7*; [73]) and trait rumination using the *Rumination Response Scale* (*RRS*; [74]).

Patients are rated for their global severity of illness (*Clinical Global Impression-Severity; CGI-S*; [75]) and its change over time (*Clinical Global Impression-Improvement; CGI-I*; [75]). Quality of life (QoL) based on aspects of *positive mood*, *vitality,* and *general interes*t is assessed using the *WHO-5 well-being index* [76]. Patients’ social, occupational, and psychological functioning in their daily life is rated using the *Global Assessment of Functioning Scale* (*GAF*; [77]).

CC functions are measured behaviorally across a wide range of neuropsychological domains using the computerized *Adaptive Cognitive Evaluation* (*ACE*) *battery* [78]. The ACE battery includes standardized tests for different aspects of CC, modified by incorporating adaptive algorithms, immersive graphics, video tutorials, and motivating feedback on a user-friendly interface. Adaptive algorithms ensure that comparisons between individuals of different age groups, genders, races, and cultures reflect actual differences in their cognitive ability and not disparities in the testing parameters or ceiling/floor effects. In the context of the DiSCoVeR trial, we assess the following CC functions: *inhibitory control*, *task switching*, *multitasking*, *impulsivity*, *sustained attention*, and *working memory capacity*.

Intrinsic motivation for game play and expectancy of treatment outcome are assessed after the fifth session on site and at post-intervention using the *Intrinsic Motivation Inventory* (*IMI*; [79, 80]) and the *Credibility/Expectancy Questionnaire* (*CEQ* [81]*;*).

Two additional assessments are given during the baseline visit only. The self-rated *Social Network Index* (*SNI*; [82]) is used to assess the number of high-contact roles (network diversity), the number of people in a social network, and the number of embedded networks. The self-reported *Childhood Trauma Questionnaire* (*CTQ*; [83]) is used to retrospectively assess aversive childhood experiences.

### Study endpoints

The *primary* endpoint of the study is the *feasibility* of the combined intervention as assessed at the post-intervention visit (V4), except in case of compensation sessions, where *feasibility* will be assessed at the end of treatment. To test *feasibility*, we use the following quantitative definition: the intervention is feasible if the probability of a study participant to successfully apply a number of at least 20 out of 30 sessions per protocol exceeds 50%, i.e., it is more likely to receive an adequate dosage of treatment sessions. The threshold of 20 out of 30 treatment sessions is largely based on previous trials of tDCS in MDD, i.e., being close to the tDCS session number in the ELECT TDCS trial and at a higher number of sessions compared to the SELECT trial (22 sessions and 12 sessions, respectively; [26, 27]) to prevent an insufficient dosage of the intervention in this trial. To test this hypothesis, we will determine the quotient of participants successfully completing the intervention according to this criterion relative to the total number of participants. This endpoint was chosen as the primary endpoint due to the novelty of our study design, in which the treatment is performed mostly in a home-based setting. The study has been powered to examine feasibility in the whole sample (i.e., both treatment arms together). In a second step we will estimate confidence intervals for each of the treatment arms separately.

The secondary endpoint is *efficacy,* measured as the change in *MADRS* scores from the baseline visit (V1) to the post-intervention visit (V4). Tertiary endpoints include *clinical response* (at least 50% reduction in the MADRS score from baseline), *remission rates* (MADRS score ≤ 10 points), and *depression relevant status,* assessed via changes in depressive and functional outcomes. Changes on all other assessments from baseline to post-treatment, including *QoL, rumination*, and *cognitive control functions* including *multitasking*, *inhibitory control* as well as *sustained attention* are also included as *tertiary* endpoints*.*

*Exploratory* endpoints include *durability of effects* (i.e., changes on all measures between baseline and follow-up), *expectation for treatment effects* (CEQ scale), *motivation* to participate in the game (IMI scale), and additional domains of cognitive control including the assessment of *WM capacity* and *task switching*.

*Finally, safety* endpoints include the occurrence of *(serious) adverse events* ((S)AEs; %) as reported by the patients and as systematically assessed using the *Comfort Rating Questionnaire* (*CRQ*; [84]), *vital signs* (blood pressure and heart rate), *body mass index*, *suicidal risk* as measured by the MADRS item 10, and change in *media use*, including daily engagement in media multitasking, as assessed by the *Media Multitasking inventory* (*MMI*; Rioja, K., Cekic, S., Bavelier, D. & Baumgartner, S. *Unraveling the link between media multitasking and sustained attention*, manuscript in preparation).

### Randomization and blinding

Participants are randomized to receive either active or control treatment following a parallel group assignment. Randomization is performed separately for each site. Patient allocation is stratified by the magnitude of the MADRS score (MADRS < 21.5 vs. MADRS ≥ 21.5). No other stratifiers are used to conserve statistical power. Within stratification, allocation is balanced in groups of four. Randomization is centrally performed by the coordinating site (HUJI) using an online randomization tool (Randomizer; https://www.randomizer.at).

Double-blinded treatment is implemented to minimize bias during the data assessment driven by potential expectation biases of raters and/or patients. Separate study personnel are responsible for the study assessments (*raters, i.e., blinded investigators*) as compared to the implementation and supervision of the treatment (*unblinded investigators* including *study operators*). Raters and patients are blind to the administered treatment condition. Raters are not in direct contact with the patients during the treatment sessions. Patients are asked to refrain from talking about their treatment (apart from experienced AEs) with the raters. Study participants are required to refrain from meeting each other before, during, and after assessments or treatments to maintain blindness. A *blinding check* is performed after the first treatment and after six weeks of intervention (V4), in which patients are asked whether they believe that they were assigned to the active or to the control condition.

### Sample size calculation

Sample size was determined based on the feasibility analysis (primary endpoint). Our primary hypothesis that the probability of completion is larger than 0.5 will be tested using a one-sided binomial test. Assuming *N* = 114 subjects, the one-sided binomial test would reject the null hypothesis (i.e., that the probability of completion is equal or less than 0.5) if the number of completers is ≥ 65 patients. This would constitute an effective completion rate of 57%. In terms of power, if the true completion rate is instead 2/3 (0.67), the probability of rejecting the null hypothesis would amount to approximately 0.98.

This sample size would also adequately power the efficacy analyzes (i.e., time x treatment interaction effect for MADRS change). Power in this analysis was estimated using a Monte-Carlo simulation of 10,000 datasets. Setting the threshold for detection at a significance level of 0.05 (one-sided), the estimated power for *N* = 114 subjects (38 enrolled per site) is 1-β = 0.91. A drop-out rate of 20% was incorporated, based on a comparable ongoing trial at the LMU Munich site [46]. Power measured the probability of detecting a significant time × treatment interaction effect. Effects were estimated using a linear mixed regression model (LMM), with time × treatment and center as fixed effects and subjects modeled as random effects. In the Monte-Carlo simulation, it was assumed that the time × treatment interaction effect was 4 points on the MADRS scale (adapted from [85]). The random effects were set at SD = 6 points for individual change (beyond baseline variability) and a standard deviation of 2.5 points was added as a center × treatment × time interaction. The assumed effect size of the interaction, after controlling for baseline, was Cohen’s *d* of 0.41 (medium effect size).

### Statistical analysis

For the analysis of the primary endpoint (feasibility and completion rate), we will estimate the probability of completion using the Binomial Fisher’s Exact Test, assuming individuals across centers are independent. Letting *P* be the probability of successful completion (week 6), we will reject the null hypothesis that *P*_(completion)_ ≤ 0.5. A confidence interval for *P*_(completion)_ will be estimated. In a second step, we will estimate the confidence intervals for each of the treatment arms separately.

The analysis of the secondary endpoint (efficacy) consists of an intention-to-treat (ITT) analysis of the change in MADRS scores from baseline to post-intervention by adjusting for inter-individual differences at baseline, sex, and center. A linear mixed regression model (LMM) with random intercepts and center, group, time, and the cross-level group x time interaction as fixed effects will be performed. The key value for significance will be the group × time interaction factor. In case of significant group × time interactions, post-hoc comparisons at each time point with correction of the type-I error probability will be conducted. Confidence intervals for mean differences and effect sizes will be computed. If the analysis at week 6 is found to be significant, an analysis of change over 10 weeks will be performed as a final analysis step concerning the secondary endpoint. All tertiary endpoints will be similarly analyzed using LMMs. Logistic regression will be used to analyze discrete tertiary endpoints (i.e., response and remission rates).

There will be no imputation in the ITT analyzes as the LMMs can handle missing values without excluding entire cases from the analysis. As a sensitivity analysis, LMMs can be computed using state-of-the-art multiple imputation of missing values. At 50% of subjects having completed the study, a planned interim analysis will assess safety and feasibility outcomes. The respective results will be shared with our SMB and published, but will not impact on the conduct of the study, unless major ethical concerns arise.

### Good clinical practice (GCP)/laws and regulations

All study-related procedures are carried out in accordance with the Declaration of Helsinki and the guidelines for Good Clinical Practice. The DiSCoVeR consortium confirms that research will be conducted in accordance with relevant local or national regulations of the country it is conducted in and shall be subject to prior authorization of the project by official ethics committees of the partner universities.

### Ethics approval and preregistration of the study

Ethical approvals were obtained at all three clinical study sites (LMU, Hadassah, RSU) before the trial initiation. The study was registered at clinicaltrials.gov (Trial identifier: NCT04953208).

## Discussion

The DiSCoVeR trial is a multi-site, double-blind RCT, which investigates feasibility, safety, and efficacy of a highly innovative therapeutic intervention for MDD, combining an action-like video game (LoH) with concurrent tDCS in a home-treatment setting. Methodologically, this novel intervention extends beyond current NIBS treatment strategies by employing newly developed methods, i.e., a video game targeting attentional control through action mechanics suitable for daily intervention from home and novel methods for precise electrode positioning. The intervention is integrated into one tablet-based application, which allows its delivery within a digital trial. To our knowledge, the DiSCoVeR trial is the first study investigating this cutting-edge therapeutic principle in MDD. Due to the novelty of its design, the DiSCoVeR trial is conducted as a two-arm study, testing the feasibility and efficacy of a double active intervention (active tDCS + LoH video game) in comparison with a double control condition (sham tDCS + LoH_control video game).

The concept of combining NIBS and behavioral interventions or cognitive training has been proposed earlier based on preclinical models [24, 86], and has been investigated for motor rehabilitation in stroke research [87, 88], as well as in more complex behavioral paradigms using single tasks or whole psychotherapy sessions [51, 89]. Psychotherapy represents an intervention of extremely high complexity involving a wide array of brain functions, and a very recent randomized controlled clinical trial combining tDCS and group CBT failed to show a superior efficacy of the combined treatment over CBT plus sham or CBT alone [47]. In contrast, few studies have reported synergistic effects of tDCS along with CCT in patients with MDD [49, 90, 91], however, results have been mixed (e.g., [92]). Specifically, a synergy between the paced auditory serial addition task (PASAT) and tDCS was observed in healthy subjects [51], but not in MDD patients [92], as both conditions, i.e., the PASAT plus active tDCS and the PASAT plus sham tDCS exerted antidepressant effects in MDD. Dose–response relationships i.e., schedules of combined sessions over time or stimulation intensity have not been established yet and are largely unexplored. Additionally, these mixed findings potentially indicate differences in underlying cognitive processes and related DLPFC engagement during the PASAT between healthy subjects and MDD patients [93]. On a behavioral level, the PASAT targets WM updating in a uniform and continuous manner [94] which may be differentially engaging for healthy participants compared to MDD patients. This may also potentially lead to group differences in terms of synergy between PASAT and tDCS. An alternative explanation for this difference could be that pre-existing changes in the function of DLPFC in MDD [95] may compromise the capability for neuromodulation in the respective regions. The DiSCoVeR trial attempts to overcome these potential limitations by (i) using a particularly engaging action-like video game that targets both attentional control and executive functions in a variety of contexts [57], (ii) administering a high dose of five weekly sessions over a period of six weeks, and (iii) using a bifrontal tDCS protocol similar to a protocol that has previously reported to exert antidepressant efficacy [27].

The DiSCoVeR trial is topical due to its development toward home-treatment, providing a remotely supervised domestic intervention beyond institutionalized or clinical care. To date, only few studies have investigated the feasibility and tolerability of remotely applied supervised tDCS at home [96–101]. The self-administered delivery of the intervention in the DiSCoVeR trial is enabled using a novel electrode-positioning algorithm incorporated into the home-application capturing individual facial key points, as well as a marker placed on the cap for control and adjustment of the electrode positioning. Previous studies have shown that it is possible to train the participants on how to place the electrodes embedded in a cap by themselves at home [61]. Using the integrated tablet camera, the algorithm supports the patients with real-time feedback to place the cap as instructed, ensuring the correct stimulation of the desired brain regions, and monitors the accuracy of the cap position. Though tDCS is a non-focal NIBS method, the importance of accurate electrode positioning has been shown by intracranial measurements of electric fields (e-fields) in surgical epilepsy patients as well as by computational modeling of e-field distribution [102, 103] and Opitz and colleagues [102] recommended electrode placement accuracy to be below 1 cm for reliable tDCS application across sessions. Demonstrating the feasibility, safety, and efficacy of the home-treatment while allowing for real-time remote control of treatment parameters could expand the spectrum of treatment options for patients with MDD significantly, as such home-treatment could be integrated into activities of daily life more naturally [104]. Additionally, it may help to overcome barriers that often interfere with treatment compliance, including difficulties to attend frequent on-site visits, an issue recently amplified by the COVID-19 pandemic.

Despite its strengths, the protocol of the DiSCoVeR trial has limitations that should be noted. First, the DiSCoVeR trial includes only two treatment arms: active vs. control. Given that each treatment arm includes a combination of two novel treatments (tDCS and video game), such a design will generate only preliminary proof-of-principle data of the combined treatment approach. Thus, little information will be generated regarding the effects of the separate treatment components of the combined therapeutic approach, independently of synergistic effects. In this sense, the DiSCoVeR trial will power a larger RCT, e.g., employing a 2 × 2 factorial or 4 arms design, which will allow a direct assessment of each of the treatment components in isolation. Second, since patients need to be instructed on how to operate the technology, some of the investigators including study operators, who do not conduct any study ratings, cannot be blinded. This is a common problem in studies involving behavioral interventions. To overcome any potential biases from investigators, raters in the DiSCoVeR trial remain blind to the treatment assignments of participants to avoid rater effects.

In sum, the DiSCoVeR trial could expand the range of treatment options for MDD by demonstrating the feasibility of this new neuromodulation enhanced intervention and by providing first data on the efficacy and safety of a novel home-based treatment for MDD with high scalability. Such a novel digital intervention could ultimately help overcome current barriers to mental health care, like low accessibility of non-digital psychotherapy and pharmacology treatment resistance, and represents a promising avenue toward guided, stratified or even personalized psychiatric care.

## Supplementary Information

Below is the link to the electronic supplementary material.

Supplementary file1 (PDF 273 KB)
