# Supplementary material for: Examining the synergistic effects of a cognitive control video game and a home-based, self-administered non-invasive brain stimulation on alleviating depression: the DiSCoVeR trial protocol
Source: Eur Arch Psychiatry Clin Neurosci. 2022 Oct 22;273(1):85–98. doi: 10.1007/s00406-022-01464-y (PMC9589526; doi:10.1007/s00406-022-01464-y)
Supplement: Supplementary file 1 — Supplementary file1 (PDF 273 KB) [file 406_2022_1464_MOESM1_ESM.pdf]

Supplementary Information

Article title: Examining the synergistic effects of a cognitive control video game and a home-based, self-administered non-invasive brain stimulation on alleviating depression The DiSCoVeR trial protocol.

Journal: European archives of Psychiatry and Clinical Neuroscience

Authors: Esther Dechantsreiter*, Frank Padberg*, Alon Morash, Ulrike Kumpf, Arthur Nguyen, Zeno Menestrina, Fabienne Windel, Gerrit Burkhardt, Stephan Goerigk, Takuya Morishita, Aldo Soldini, Shira Ahissar, Tamar Cohen, Angela Pasqualotto, Linda Rubene, Liene Konosonoka, Daniel Keeser Peter Zill, Razan Assi, Rémy Gardier, Roser Viñals, Jean-Philippe Thiran, Ronen Segman, Yuval Benjamini, Omer Bonne, Friedhelm Christoph Hummel, Daphne Bavelier, Elmars Rancans, Mor Nahum

* Co-first authorship

Corresponding author:

Frank Padberg

padberg@med.uni-muenchen.de

Department of Psychiatry and Psychotherapy, Ludwig Maximilians University Munich, Nussbaumstr. 7, 80336 Munich, Germany

Table of Content:

Online Resource 1 Medication at study inclusion and concomitant treatment

Online Resource 2 Time & Events Table

Online Resource 3 Adjunctive Assessments

Online Resource 1 Medication at study inclusion and concomitant treatment

Selective serotonin reuptake inhibitors (SSRI): escitalopram, citalopram, sertraline, paroxetine, fluvoxamine, fluoxetine; Selective serotonin noradrenaline reuptake inhibitors (SSNRI): duloxetine, venlafaxine, milnacipran; Others: mirtazapine, agomelatine, vortioxetine, bupropion. Combined treatment strategies of the aforementioned medication with quetiapine (up to a dose of 300 mg/d) or with aripiprazole (up to a dose of 5 mg/d), is allowed, if stable for at least 4 weeks. Combined treatment with lithium is allowed, if stable for at least 3 months. Antidepressant medication, mood stabilizers, and antipsychotics other than stated above, are not allowed throughout the acute study phase. Only the following rescue medication may be prescribed during the acute study phase: zopiclone (on demand up to 7.5 mg/day orally), zolpidem (on demand up to 12.5 mg/day orally), benzodiazepines (on demand up to 2.0 mg/day, equivalent to a dose of lorazepam), quetiapine (on demand up to a dose of 75 mg/day), ibuprofen, paracetamol, acetylsalicylic acid (ASA) for treatment of local pain, dental pain or headaches is allowed, as necessary.

Online Resource 2 Time & Events Table

	Pre-treatment		Intervention period																														Follow-up
**Week**	**-1**		**1**					**2**					**3**					**4**					**5**					**6**					**10**
**Study visit**	**S (V0)**	**B (V1)**										**V2**										**V3**										**V4**	**V5**
**Day**	-14/-5	-4/-1	1	2	3	4	5					12										26										40	68
**Screening, baseline rating and administrative procedures**																																	
Informed consent	√																																
In-/exclusion criteria	√																																
MINI	√																																
Demographic data + family history of psychiatric illness	√																																
EHI	√																																
FTND	√																																
HDRS-17	√																																
General medical and psychiatric history	√																																
Antidepressant Treatment History – ATHF	√																																
Physical and Neurological Examination	√																															√	
Pregnancy test[a]	√																																
SNI		√																															
CTQ		√																															
Concomitant treatment[b]	√	√					√					√					√					√					√					√	√
Randomization		√																															
Blinding check			√																													√	
Media multitasking index (MMI)		√																														√	√
Gaming Disorder Test	√																																
Post-Evaluation Questionnaire																																√	
**Endpoints**																																	
**Primary Endpoint**																																	
Feasibility (primary)																																√	
**Secondary Endpoint**																																	
MADRS	√	√										√										√										√	√
**Tertiary Endpoints**																																	
PHQ-9		√										√										√										√	√
BDI-II		√										√										√										√	√
CGI-S		√										√										√										√	√
CGI-I												√										√										√	√
WHO-5		√																														√	√
GAF		√										√										√										√	√
ACE battery		√																														√	√
RRS		√										√										√										√	√
**Exploratory Endpoints**																																	
GAD-7		√										√										√										√	√
IMI							√																									√	
CEQ							√																									√	
**Safety measures**																																	
Vital signs, BMI [c]	√	√																														√	
CRQ			√	√	√	√	√	√	√	√	√	√	√	√	√	√	√	√	√	√	√	√	√	√	√	√	√	√	√	√	√	√	
Suicidality (MADRS 10)	√	√										√										√										√	
**Adjunctive measures**																																	
Biological testing		√																														√	√
Multimodal fMRI		√																														√	√

[a]For females of child-bearing potential [b]Recording of medication as well as psychotherapeutic intervention, duration, and frequency; [c]Vital signs: Pulse and systolic and diastolic blood pressure; Basic Somatic Information: BMI and Head Size

Instruments und Scales: MINI - International Neuropsychiatric Inventory; EHI - Edinburgh Handedness Inventory; FTND - Fagerström Test for Nicotine Dependence; HDRS-17 - Hamilton Depression Rating Scale- 17 questions; ATHF - Antidepressant Treatment History Form; SNI - Social Network Index, CTQ - Childhood Trauma Questionnaire, MADRS - Montgomery-Asberg Depression Rating Skala; PHQ-9 - Patient Health Questionnaire 9; RRS - Rumination Response Scale; CGI-S - Clinical Global Impression-Severity; CGI-I-Clinical global impression - Improvement Scale; GAD-7 - Generalized Anxiety Disorder 7-item; WHO-5 - World Health Organization Five Well-Being Index; GAF – Global Assessment of Functioning; ACE battery - Adaptive Cognitive Evaluation battery; IMI - Intrinsic Motivation Inventory; CEQ – Credibility/Expectancy questionnaire; BMI - Body Mass Index; CRQ - Comfort Rating Questionnaire

Online Resource 3 Adjunctive Assessments

Salivary predictors of treatment response and epigenetic modification during treatment

Depressive disorders are significantly associated with hereditary factors [1]. Previous research has examined markers of antidepressant response at the level of DNA base variation, epigenetics, and gene expression and has related them to several candidate transmitter systems, neuromodulator systems, and neurotrophic as well as to their respective pathologies. Among others, literature suggests focusing on the serotonin transporter gene (5-HTTLPR), the serotonin receptor 1A and 2A genes (HTR1A, HTR2A), the tryptophan hydroxylase-2 gene (TPH2), the monoamineoxidase oxidase A gene (MAOA), the catechyl-o-methyl transferase gene (COMT), the brain derived neurotrophic factor gene (BDNF), the apolipoprotein E4 gene, and on genes that underlie stress hormone functions (CRHR1 and FKBP5) and attachment (oxytocin receptor gene, OXTR; [2-4]), but with partially conflicting results. In addition to such candidates, that were based on a priori knowledge of known antidepressant drug response targets, more recently, a large genome wide association study (GWAS) revealed 44 MDD risk genes that pass genome wide significance levels [5].

There is also increasing interest in epigenetic mechanisms as predictors for treatment response. Epigenetic mechanisms have been suggested as important contributors to the pathogenesis of depression due to their mediating role on environmental influences that could alter one's vulnerability for the disease. In this context a recent study showed a continuous increase in COMT promoter DNA methylation after a stressful cognitive task, which was correlated with higher saliva cortisol levels [6]. This lasting effect was suppressed by concurrent activity-enhancing anodal tDCS to the dorsolateral prefrontal cortex. These findings support the significance of gene-specific DNA methylation in peripheral tissues like saliva or blood as potential biomarkers for stress-related effects. Moreover, they suggest alternative molecular mechanisms possibly involved in longer-lasting behavioral effects of tDCS. In the DiSCoVeR project patients optionally provide salivary samples before (V1) and after treatment (V4 and V5). At these three time points, saliva samples are collected using OG-500 saliva tubes (Oragene) to assess DNA methylation at specific sites in several previously linked candidate genes encoding the glucocorticoid receptor, FKBP-5, oxytocin,

and its receptor, BDNF, key monoaminergic enzymes receptors and transporters, as well as several MDD risk genes discovered in the recent large scale MDD GWAS [5]. DNA will be prepared following a standard procedure. The determination of the DNA-methylation pattern in candidate genes will be carried out by bisulfite sequencing using commercially available PyroMark CpG assays on the PyroMark Q48-System (Qiagen). DNA will be bisulfite treated with the EpiTect Bisulfite Kit (Qiagen). While the current sample size does not allow for a meaningful evaluation of genetic variant associations, we will perform an exploratory investigation of DNA methylation patterns and secreted salivary hormones and cytokines that may be predictive of the clinical response to tDCS. All laboratory procedures are carried out blind to case control status.

Multimodal imaging paradigm

At two clinical study sites (Hadassha, Israel; LMU, Germany) patients optionally participate in an additional multiparametric magnetic resonance imaging (MRI) paradigm. The overall aim of this assessment is to gain a deeper mechanistic understanding of the intervention conducted within the trial. Synergistic effects of the intervention are investigated on a functional and structural level. Additionally, tDCS-induced electric fields (e-fields) are simulated based on the patient-individual head anatomy.

At three time points (V1, V4 and V5), structural MRI data (T1, T2), field maps, and resting state functional MRI (rsfMRI) are obtained. Data acquisition is carried out using a 3T Siemens MAGNETOM Skyra 3T scanner at the ELSC Neuroimaging Unit in Israel and using a Siemens MAGNETOM Prisma 3T Research MRI scanner in Germany.

At the LMU trial site, transcranial magnetic stimulation (TMS) is administered along with MRI image acquisition (*online-TMS*) at baseline, after six weeks of intervention, and at follow-up. Only single experimental TMS sessions are applied expecting no therapeutic effects. Online-TMS is conducted to investigate the individual capability for the functional modulation of prefrontal target areas [7,8]. For this purpose, TMS-pulses below the therapeutic range of TMS interventions are applied. TMS-induced modulations of local and global neuronal activity in prefrontal tDCS/CC target regions are determined by capturing changes in the BOLD (*blood oxygenation level dependent*) signal. BOLD-signal changes detected under the TMS coil are assumed to reflect an intensity-dependent increase in local neuronal activity [9], Given that BOLD signals are only transient [10], fMRI data is recorded during TMS over the left DLPFC simultaneously. A control group consisting of age- and gender-matched healthy volunteers is assessed once with the same online-TMS paradigm.

References

1. Smoller JW (2016) The Genetics of Stress-Related Disorders: PTSD, Depression, and Anxiety Disorders. Neuropsychopharmacology 41:297–319. https://doi.org/10.1038/npp.2015.266

2. Gong Q, He Y (2015) Depression, neuroimaging and connectomics: a selective overview. Biol Psychiatry 77:223–235. https://doi.org/10.1016/j.biopsych.2014.08.009

3. Won E, Ham B-J (2016) Imaging genetics studies on monoaminergic genes in major depressive disorder. Prog Neuropsychopharmacol Biol Psychiatry 64:311–319. https://doi.org/10.1016/j.pnpbp.2015.03.014

4. Nieratschker V, Kiefer C, Giel K, et al (2015) The COMT Val/Met polymorphism modulates effects of tDCS on response inhibition. Brain Stimul 8:283–288. https://doi.org/10.1016/j.brs.2014.11.009

5. Wray NR, Ripke S, Mattheisen M, et al (2018) Genome-wide association analyses identify 44 risk variants and refine the genetic architecture of major depression. Nat Genet 50:668–681. https://doi.org/10.1038/s41588-018-0090-3

6. Wiegand A, Blickle A, Brückmann C, et al (2021) Dynamic DNA Methylation Changes in the COMT Gene Promoter Region in Response to Mental Stress and Its Modulation by Transcranial Direct Current Stimulation. Biomolecules 11.: https://doi.org/10.3390/biom11111726

7. Bergmann TO, Varatheeswaran R, Hanlon CA, et al (2021) Concurrent TMS-fMRI for causal network perturbation and proof of target engagement. Neuroimage 237:118093. https://doi.org/10.1016/j.neuroimage.2021.118093

8. Mizutani-Tiebel Y, Chang K-Y, Tik M, et al (2021) Systematic review of interleaved TMS-fMRI – overview of methodological differences and sources of bias. Brain Stimulation 14:1684

9. Navarro de Lara LI, Tik M, Woletz M, et al (2017) High-sensitivity TMS/fMRI of the Human Motor Cortex Using a Dedicated Multichannel MR Coil. Neuroimage 150:262–269. https://doi.org/10.1016/j.neuroimage.2017.02.062

10. Thut G, Pascual-Leone A (2010) A review of combined TMS-EEG studies to characterize lasting effects of repetitive TMS and assess their usefulness in cognitive and clinical neuroscience. Brain Topogr 22:219–232. https://doi.org/10.1007/s10548-009-0115-4
